# Emerging Role of Calcium-Activated Potassium Channel in the Regulation of Cell Viability Following Potassium Ions Challenge in HEK293 Cells and Pharmacological Modulation

**DOI:** 10.1371/journal.pone.0069551

**Published:** 2013-07-16

**Authors:** Domenico Tricarico, Antonietta Mele, Sara Calzolaro, Gianluigi Cannone, Giulia Maria Camerino, Maria Maddalena Dinardo, Ramon Latorre, Diana Conte Camerino

**Affiliations:** 1 Department of Pharmacy-Drug-Science, University of Bari, Bari, Italy; 2 Centro de Neurociencias de Valparaíso, Facultad de Ciencias, Universidad de Valparaiso, Valparaíso, Chile; University of Houston, United States of America

## Abstract

Emerging evidences suggest that Ca^2+^activated-K^+^-(BK) channel is involved in the regulation of cell viability. The changes of the cell viability observed under hyperkalemia (15 mEq/L) or hypokalemia (0.55 mEq/L) conditions were investigated in HEK293 cells expressing the hslo subunit (hslo-HEK293) in the presence or absence of BK channel modulators. The BK channel openers(10^-11^-10^-3^M) were: acetazolamide(ACTZ), Dichlorphenamide(DCP), methazolamide(MTZ), bendroflumethiazide(BFT), ethoxzolamide(ETX), hydrochlorthiazide(HCT), quercetin(QUERC), resveratrol(RESV) and NS1619; and the BK channel blockers(2x10^-7^M-5x10^-3^M) were: tetraethylammonium(TEA), iberiotoxin(IbTx) and charybdotoxin(ChTX). Experiments on cell viability and channel currents were performed using cell counting kit-8 and patch-clamp techniques, respectively. Hslo whole-cell current was potentiated by BK channel openers with different potency and efficacy in hslo-HEK293. The efficacy ranking of the openers at -60 mV(Vm) was BFT> ACTZ >DCP ≥RESV≥ ETX> NS1619> MTZ≥ QUERC; HCT was not effective. Cell viability after 24 h of incubation under hyperkalemia was enhanced by 82+6% and 33+7% in hslo-HEK293 cells and HEK293 cells, respectively. IbTx, ChTX and TEA enhanced cell viability in hslo-HEK293. BK openers prevented the enhancement of the cell viability induced by hyperkalemia or IbTx in hslo-HEK293 showing an efficacy which was comparable with that observed as BK openers. BK channel modulators failed to affect cell currents and viability under hyperkalemia conditions in the absence of hslo subunit. In contrast, under hypokalemia cell viability was reduced by -22+4% and -23+6% in hslo-HEK293 and HEK293 cells, respectively; the BK channel modulators failed to affect this parameter in these cells. In conclusion, BK channel regulates cell viability under hyperkalemia but not hypokalemia conditions. BFT and ACTZ were the most potent drugs either in activating the BK current and in preventing the cell proliferation induced by hyperkalemia. These findings may have relevance in disorders associated with abnormal K^+^ ion homeostasis including periodic paralysis and myotonia.

## Introduction

Potassium ions regulate inflammation, oxidative stress, vascular biology and blood pressure, the excitability of the cells, exerting beneficial effects on different tissues [1–3]. Abnormalities in the serum potassium ion levels are associated with acquired and congenital diseases affecting several apparatus including skeletal muscle [4].

Severe hyperkalemia characterizes the hyperkalemic renal tubular Acidosis (type IV), mineralocorticoid deficiency (hypoaldosteronism states) as well as tumor lysis syndrome, rhabdomyolysis, marked leucocytosis and thrombocytosis, trauma and burns [5]. Disease progression and increased hearth mortality are observed in chronic kidney disease under hypokalemia or hyperkalemia conditions and these effects are gender and race dependent [6]. Severe nephropathy with renal interstitial fibrosis and ventricular hypertrophy are seen in human patients under hyperkalemia states [7,8].

Marked variations in serum potassium concentration characterize the primary periodic paralyses (PP) which are rare autosomal-dominant disorders affecting neuromuscular apparatus characterized by episodes of muscle weakness and paralysis. The primary PP is hyperkalemic periodic paralysis, hypokalemic periodic paralysis and Andersen’s syndrome [9]. Other related disorders are the thyrotoxic periodic paralysis associated with thyrotoxicosis. The familial periodic paralysis and thyrotoxic periodic paralysis are linked to mutations in the skeletal muscle sodium, calcium or potassium channel genes associated with muscle fiber depolarization and un-excitability [9–12].

Besides the short-term arrhythmogenic effects of hypo- and hyperkalemia, abnormalities of potassium ion homeostasis have a clear negative impact on clinical outcomes in neuromuscular disorders but the pathomechanisms associated with hyperkalemia or hypokalemia conditions are not well understood [13]. Vacuole myopathy and t-tubule aggregates characterize muscle biopsies of hypoPP patients and K-depleted rats, a not genetic animal model of the disease [9,14]. Progressive muscular atrophy and permanent weakness were found in hypoPP patients carrying the CACNA1S gene mutations [15]. In Andersen’s Syndrome, the loss of function mutations of KCNJ2 gene encoding for the Kir2.1 is associated with arrhythmias, muscle weakness and skeletal muscle dysmorphisms as demonstrated in the Kir2.1 knockout mice, which exhibits a narrow maxilla and complete cleft of the secondary palate that may mimic the facial dysmorphology, observed in humans [9,16]. In this case, the loss of function mutation of the Kir2.1 channel is associated with an abnormal cell proliferation that reduces the cell viability explaining the dysmorphology characterizing the phenotype [16,17]. The Kir2.1 channel is indeed active in differentiating cells inducing hyperpolarisation and setting the *E*m in a range where calcium ions can enter the myoblasts through calcium -permeable channels, which promotes the differentiation and fusion of myoblasts.

Abnormal response to potassium ions is also observed in the potassium aggravated myotonia which is a neuromuscular disorder characterized by fibers hyperexcitability, myotonic contraction and the spontaneous electrical activity of the fibers known as after-discharge [18,19]. Myotonia is genetically linked to the loss of function mutation of the sarcolemma chloride channel or gain of function mutations of the sarcolemma sodium channel, which explains most of the symptoms of the disease. In myotonia, the excessive efflux of potassium ions, which accumulates into the T-tubules of the fibers during trains of action potentials, leads to paralysis [19,20]. The myotonic phenotype in humans and animals consists of severe and invalidating dysmorphology due to skeletal muscle hypertrophy suggesting that abnormal cell viability is also occurring in these cases.

Emerging evidences suggest that the calcium-activated potassium- channel (BK) channel plays a role in cell viability in different cell types including osteoblasts, vascular smooth muscle cells and in cell lines expressing the recombinant channel subunits [21–23]. BK channels are composed by the alpha subunit encoded by one gene (slo1/KCNMA1) assembled as tetramer and transmembrane beta subunits (beta1−4). The alpha subunit of BK channels may assemble with beta−subunits with 1:1 stoichiometry enhancing the calcium sensitivity, favoring the trafficking into the membrane and modulating the pharmacological responses. The alpha, alpha+beta 1, alpha+beta 3 or beta 4 mimics the skeletal muscle, vascular smooth muscle and neuronal BK channels, respectively [24].

It has been recently shown that high glucose enhances HEK293 cell viability by inhibition of cloned BK channel subunits hslo + beta 1 [22]. The BK channel openers NS1619 or tamoxifen significantly induced apoptosis reducing cell viability in cells expressing the combination of the hslo + beta 1 subunits under hyperglycemia conditions suggesting that cloned BK channel directly regulates apoptosis and proliferation of HEK293 cell. It has been proposed that this effect may have a role in the diabetic vascular dysfunction associated with a vascular wall hypertrophy [22]. However, the potential role of the hslo subunit in regulating the cell viability in response to changes of the external potassium ion concentration has never been investigated. This may have relevance in the pathophysiology of neuromuscular disorders associated with abnormal potassium ion homeostasis such as the PP and myotonia.

The drug treatment of the hyperkalemia and hypokalemia in PP and related neuromuscular disorders is achieved by the administration of the benzothiazide diuretics. But the most effective medications for the prevention of attacks in both disorders remain the use of acetazolamide and dichlorphenamide which ameliorate paralysis reducing the frequency of the attacks. One of the main mechanisms responsible for the therapeutic effects of these drugs is related to the capability to open the muscular BK channels [25–27]. These drugs activate BK channels in excised patches experiments from normal and depleted rat fibers at micromolar concentrations indicating that the observed actions are due to a direct interaction of the molecules with the channel subunits in the isolated membrane. However the effects of acetazolamide and dichlorphenamide or benzothiazide diuretics on cell viability are not known.

**Figure 1:**
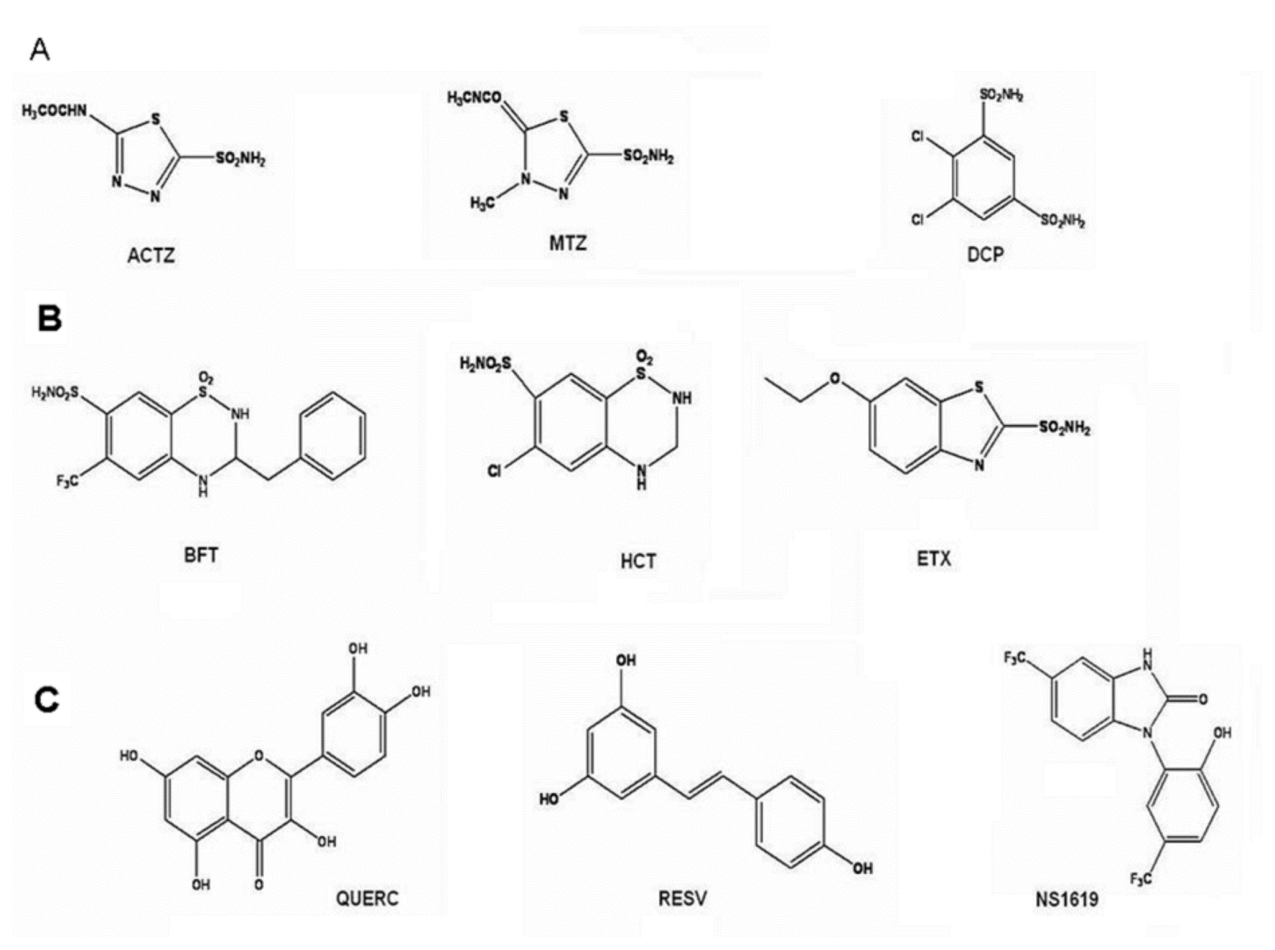
Molecular structures of the compounds under investigation. (A) acetazolamide (ACTZ), methazolamide (MTZ), dichlorphenamide (DCP). (B) bendroflumethiazide (BFT), hydrochlorthiazide (HCT), ethoxzolamide (ETX). (C) flavonoids: quercetin (QUERC), resveratrol (RESV), and the benzimidazolone (NS1619).

In the present work the potential role of the recombinant human alpha subunit (hslo) expressed in HEK293 cell line in regulating the cellular viability in response to changes of the external potassium ion concentration was investigated. The alpha subunit represents the mayor subunit expressed in skeletal muscle in the absence of beta subunits [24]. The capability of several BK channel openers to modulate the cellular viability and BK channel currents were investigated. The BK openers under investigation were : the sulphonamides drugs as acetazolamide (ACTZ), dichlorphenamide (DCP), etoxzolamide (ETX) and methazolamide (MTZ); the benzothiazides drugs as bendroflumethiazide (BFT) and hydrochlorthiazide (HCT); the flavonoids quercetin (QUERC) and resveratrol (RESV); the NS1619, a benzimidazolone molecule (Figure 1A, B, C).

## Results

### Effects of the external K^+^ ions challenge on hslo channel currents recorded in HEK293 cells and pharmacological modulation

The exposure of the hslo-HEK293 cells to increasing concentrations of external K^+^ ions significantly reduced the outward hslo current. The mean hslo current amplitude, leak subtracted, in the presence of 150x10^-3^M K^+^ ions in the cell, was -421+24 pA, -451+34 pA, -501+41 pA and -578+21 pA (p<0.05) at -60 mV (Vm), and it was 1600+34 pA, 931+31 pA(p<0.05), 399+24 pA (p<0.05) and 321+29 (p<0.05) at +30 mV (Vm) in the presence of 0.55, 5, 15 and 150 x 10^-3^M K^+^ ions in the bath solution, respectively (Number of cells = 235).

**Figure 2:**
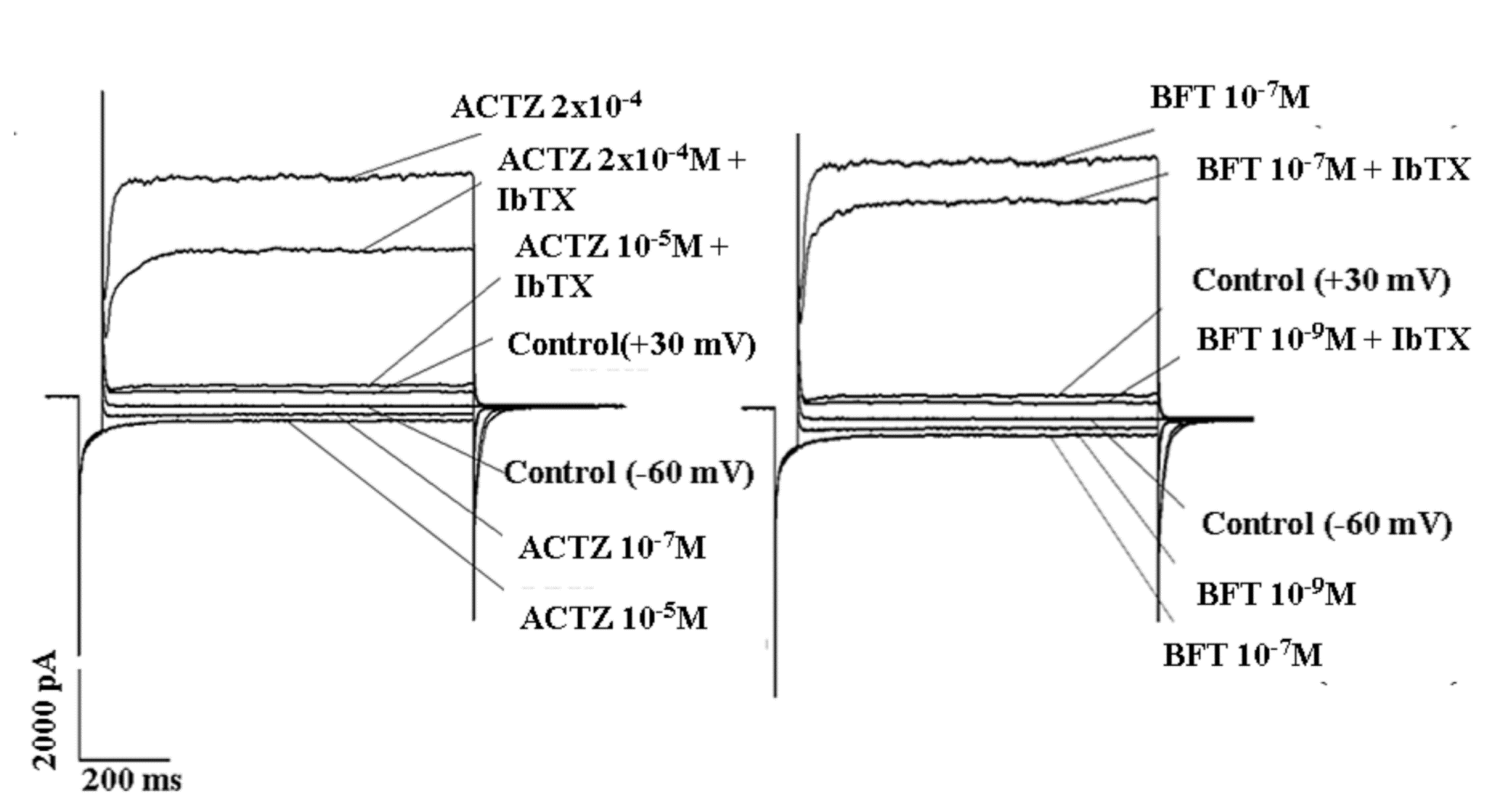
Sample traces of macroscopic whole hslo channel currents recorded in HEK293 cell in the absence (control) and in the presence of bendroflumethiazide (BFT) and acetazolamide (ACTZ). The currents were recorded in the presence of 150 x10^-3^M K^+^ ions in the pipette and 5 x10^-3^M K^+^ ions in the bath solutions, in the presence of 10^-6^ M concentration of free Ca^2+^ ions in the cell, using a voltage protocol: holding potential 0 mV; prepulse, -150 mV; test pulse, from -150 mV to + 150 mV in 10 mV steps; tail, -60 mV. The currents didn’t leak subtracted. The represented currents traces showed the effects of BFT and ACTZ on hslo channel currents at -60 mV (Vm) and + 30 mV (Vm). BFT was more effective than ACTZ in activating the hslo current at +30 mV and -60 mV (Vm). Iberiotoxin (IbTX) (4x10^-7^M) fully blocked the hslo currents activated by BFT (10^-9^M) and ACTZ(10^-5^M) tested at a low concentration. The toxin caused a partial block of the channel currents activated by high concentrations of BFT (10^-7^M) and ACTZ (2x10^-4^M).

The drugs under investigation were capable to activate the currents flowing through the hslo channel subunit expressed in HEK293 cell. BFT caused a marked enhancement of the whole hslo current at negative and positive membrane potentials as compared with ACTZ in the presence of 5x10^-3^M K^+^ ions in the bath solution (Figure 2). The exposure of the hslo-HEK293 cells to IbTX (4x10^-7^M) or ChTX (2x10^-7^M) produced a partial reduction of the drug-activated channel currents and a full block was observed at +30 mV (Vm) in the presence of a low drug concentration (Figure 2).

**Figure 3:**
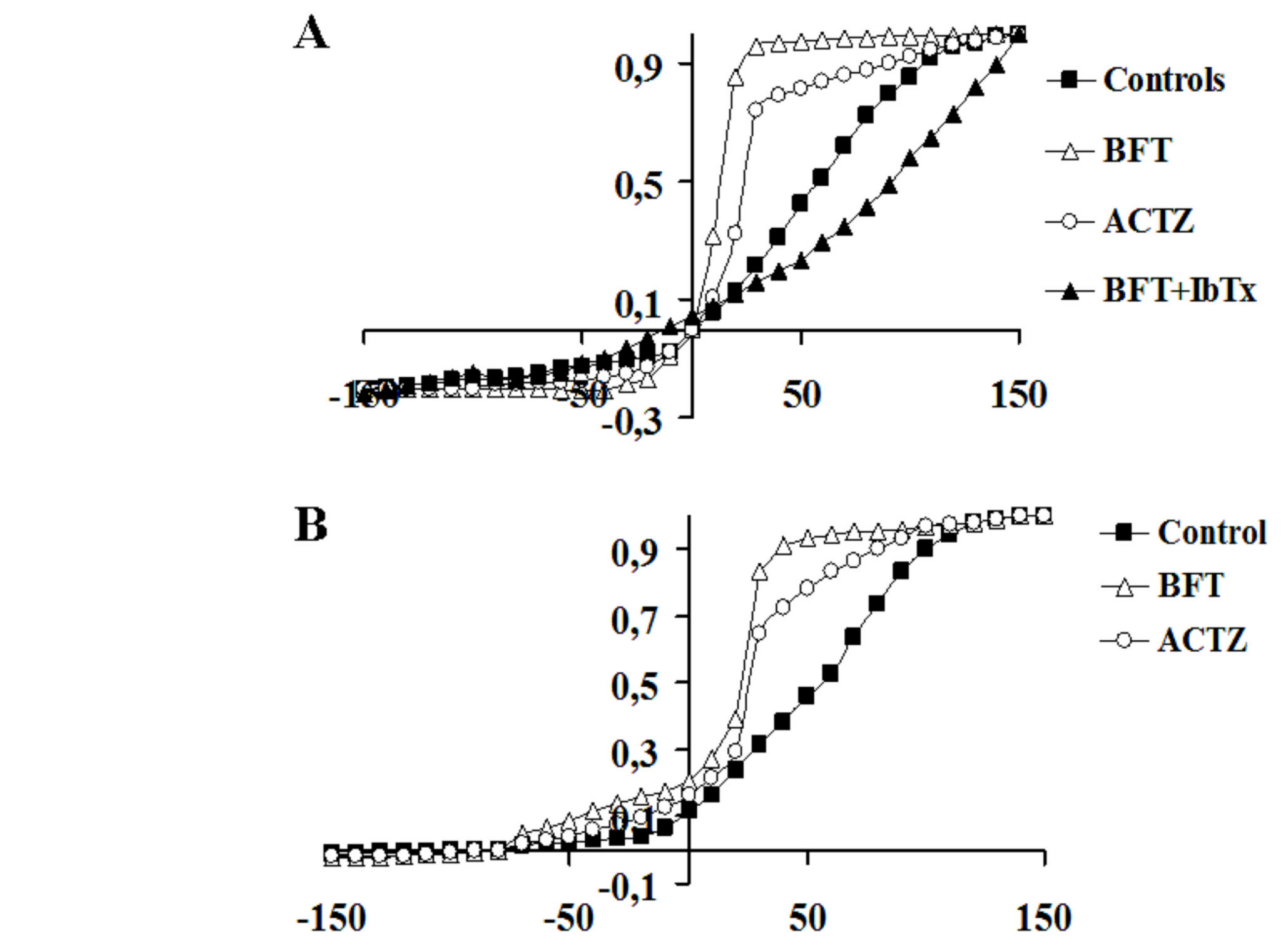
Effects of bendroflumethiazide (BFT) (10^-9^ M) and acetazolamide (ACTZ) (2x10^-4^M) on the current-voltage relationships of hslo channel current. The currents were recorded in (A) symmetrical (150 x10^-3^ KCl intracellular /150 x10^-3^ KCl extracellular) and (B) not symmetrical K^+^ ion conditions (150 x10^-3^ KCl intracellular /5 x10^-3^ KCl in the bath) in the same cell. The hslo channel currents were recorded in whole configuration in the presence of 10^-6^ M concentration of free Ca^2+^ ions in the cell using a voltage protocol: holding potential 0 mV; prepulse, -150 mV; test pulse, from -150 mV to + 150 mV in 10 mV steps; tail, -60 mV. The maximal change in the current amplitude was observed at +30 mV (Vm) and -60 mV (Vm) with BFT and ACTZ. The co-application of iberiotoxin at the 4x10^-7^M concentration and BFT to the cell at the end of the drug protocol caused a full block of the BFT activated current.

I–V relationship analysis revealed that in the presence of 150 x10^-3^M K^+^ ions in the bath solution and in the cell, BFT and ACTZ significantly enhanced inward and outward currents recorded at negative and positive membrane potentials, respectively (Figure 3A). These drugs caused a maximal change of the currents at -60 mV and +30 mV (Vm). In the presence of 5x10^-3^M K^+^ ions in the bath, BFT and ACTZ increased the outward currents at negative and positive membrane potentials (Figure 3B).

**Figure 4:**
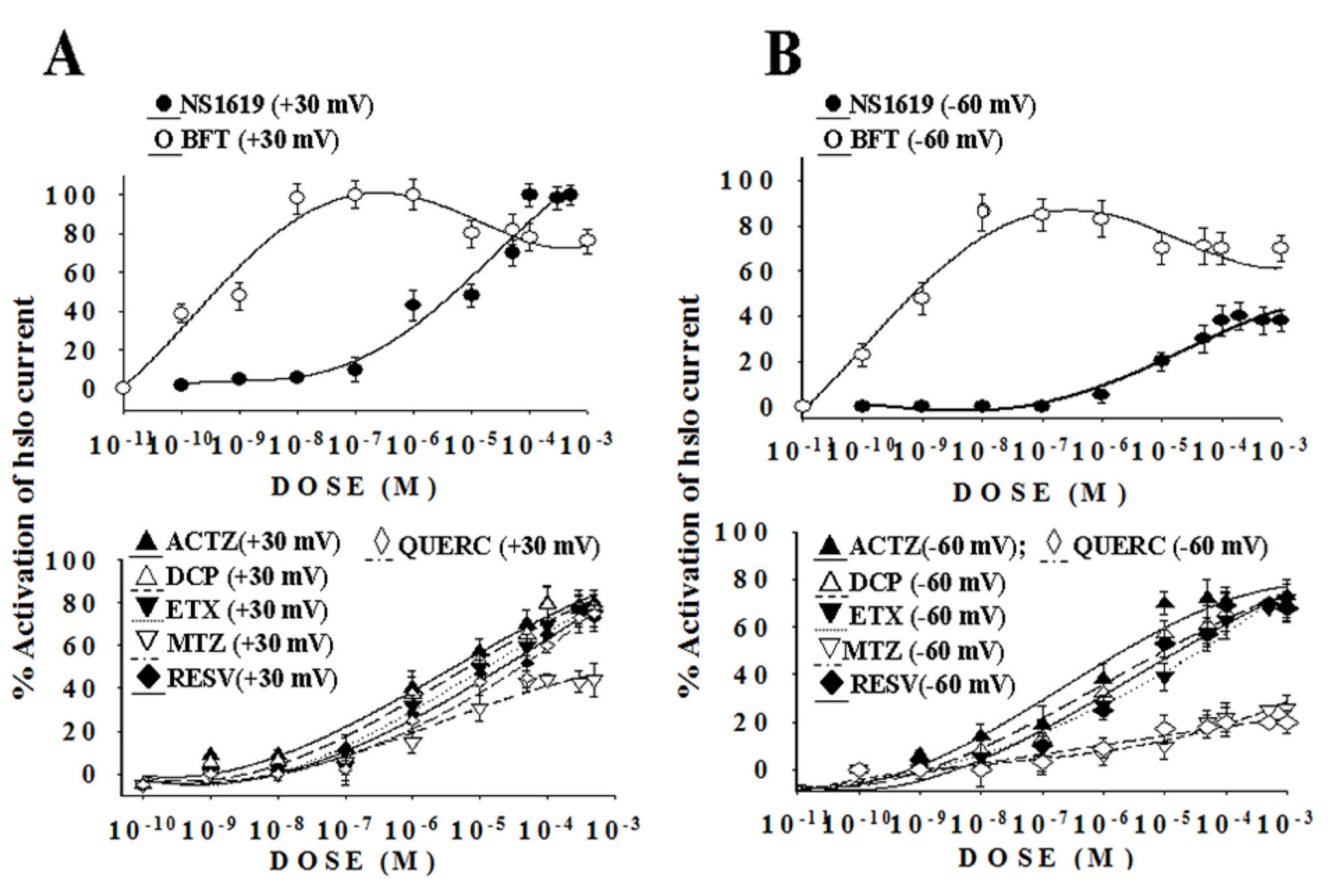
Concentration–response relationships of whole hslo channel currents vs drug concentrations under normokalemia conditions. The concentration–response relationships of NS1619, bendroflumethiazide (BFT), acetazolamide (ACTZ), dichlorphenamide (DCP), methazolamide (MTZ), ethoxzolamide (ETX), quercetin (QUERC) and resveratrol (RESV) were constructed at +30 mV (Vm) (A) and -60 mV (Vm) (B). The hslo channel currents were recorded in whole configuration in asymmetrical K^+^ ion concentrations (150 x10^-3^ KCl intracellular /5 x10^-3^ KCl extracellular), in the presence of 10^-6^ M concentration of free Ca^2+^ ions in the cell. Each experimental point represents the mean ± S.E. of the percentage activation of the hslo channel currents versus the compound concentrations of a minimum of four and a maximum of six patches. Biphasic concentration–response relationships were constructed for BFT, while monophasic concentration–response curves were constructed for all other drugs. BFT and NS1619 were the most effective hslo openers at +30 mV (mV), and BFT was the most effective and potent opener also at -60 mV (Vm) as compared with the other drugs.

Concentration–response relationships, performed in the presence of 5x10^-3^M K^+^ ions in the bath solution and 150x10^-3^M K^+^ ions in the cell, showed that the drugs under investigation activated the whole hslo channel current with different potency and efficacy (Figure 4A, B). The drug effects were reversible within 6-8 sec of washout period except for QUERC action. Significant activating actions were observed at all voltage membranes with BFT, ACTZ, DCP, ETX, RESV, NS1619, QUERC and MTZ but not observed with HCT. The concentration–response relationships of the drugs (10^-11^-10^-3^M) constructed at -60 mV and +30 mV (Vm) were fitted to the equation 1 describing the interaction of the molecules with one stimulatory site, except for BFT data. BFT molecule indeed enhanced the whole hslo currents in the nanomolar concentration range however showing a reduced capability to increase the channel current at concentration >10^-6^M, the BFT data were therefore fitted to the sum of equations 1 and 2. BFT was the most potent drug in enhancing the whole hslo currents in the range of concentrations tested, at all membrane potentials, within the investigated molecules (Figure 4A, B). We found that the threshold concentrations and EC_50a_ needed to activate the hslo channel with respect to controls at negative membrane potentials were in the sub-nanomolar concentration range for BFT, submicromolar concentrations for ACTZ, DCP, ETX and RESV and were > 10^-6^M concentrations for NS1619, MTZ, and QUERC. This caused a shift of the concentration–response relationships of BFT, ETX, DCP, ACTZ and RESV molecules to the left on the log concentration axis at -60 mV (Vm) (Figure 4B). The potency ranking of the openers expressed as EC_50a_ at -60 mV (Vm) was BFT> ACTZ> DCP>ETX>RESV > NS1619> MTZ>QUERC (Table 1). The most effective drugs in inducing the maximal channel activation (Amax_a_) were BFT, ACTZ, DCP, ETX and RESV; in the same experimental conditions NS1619, MTX and QUERC caused not more than 40% enhancement of the currents while HCT was not effective. This was confirmed using the one-way analysis of variance that allowed a multiple comparison of 8 different drug treatments performed in the same range of concentrations. This analysis showed that the calculated variance ratio for the drug treatments was higher than that theoretically tabulated at the corresponding degree of freedom indicating that the drug treatments at -60 mV (Vm) were not equal each other (p<0.05). The absolute efficacy ranking of the openers based on the variance analysis at -60 mV (Vm) was therefore BFT> ACTZ≥DCP≥ RESV≥ ETX > NS1619> MTZ≥QUERC.

**Table 1:** Fitting parameters of the concentration–response curves of BK channel openers concentrations vs whole cell hslo currents recorded at different membrane potentials in hslo-HEK293 cells.

	+30 mV (Vm)			-60 mV (Vm)		
**Compounds**	**Amax_a_/Amax_b_ (%)**	**EC_50a_/EC_50b_ (M)**	***n*_*a*_*/n*_*b*_**	**Amax_a_/Amax_b_ (%)**	**EC_50a_/EC_50b_ (M)**	***n*_*a*_*/n*_*b*_**
**NS1619**	100±21	1.1±1x10^-5^	3.23±1	38±3	5.5±1x10^-5^	0.32±0.5
**BFT**	100±10/81±11	1.2±1x10^-9^/5±1x10^-6^	1.35±0.2/-1.3±0.2	83±9/61±12	1.1±3x10^-9^/4.1±1x10^-6^	1.0±0.1/-1±0.1
**ACTZ**	80±9	1.1±1x10^-6^	1.17±0.9	73±5	1.21±2x10^-7^	0.93±0.1
**DCP**	78±8	3.39±1x10^-6^	1.25±0.8	69±6	4.3±1x10^-7^	0.81±0.1
**MTZ**	43±15	1.0±1x10^-5^	0.6±0.7	31±7	1.17±1x10^-4^	0.3±0.2
**ETX**	76±16	5.8±1.1x10^-6^	1.2±1.1	69±4	7.1±0.1x10^-7^	0.88±0.01
**QUERC**	73±10	2.21±1x10^-5^	1.65±0.3	22.8±6	1.5±0.1x10^-4^	0.49±0.01
**RESV**	76±7	7.19±1x10^-6^	1.5±1	68±9	5.5±1x10^-7^	0.97±0.01

Compounds are NS1619, bendroflumethiazide (BFT), acetazolamide (ACTZ), dichlorphenamide (DCP), methazolamide (MTZ), ethoxzolamide (ETX), quercetin (QUERC) and resveratrol (RESV). Amax_a_ and Amax_b_ are the maximal activation of the whole cell hslo currents produced by the compounds; EC_50a_ and EC_50b_ are the concentrations of the drug needed to enhance the current by 50%; *n*_*a*_ and *n*_*b*_ are slope factors of the concentration–response relationships.

The capability of the drugs to maximally activate the hslo channel was enhanced by patch depolarization (Figure 4A). The absolute efficacy ranking of the openers based on the analysis of variance at +30 mV (Vm) was BFT> NS1619> ACTZ>DCP>ETX>RESV>QUERC> MTZ which was different in respect to that observed at -60 mV(Vm). The potency ranking of the openers expressed as EC_50a_ at the same voltage membrane was BFT> ACTZ>DCP>ETX >RESV> NS1619>QUERC>MTZ which was similar to that observed at -60 mV(Vm) (Table 1).

HCT was not effective as opener of the hslo channel currents in the range of concentrations tested at negative or positive membrane potentials.

The Hill slopes were significantly higher than unit at positive voltages for all effective drugs, while at negative voltage the Hill slopes for most of the drugs were less than unity. These results suggest changes in either the stoichiometry of the binding reaction of the molecules with channel subunit with depolarization or a modification of the cooperation between sites (Table 1).

**Figure 5:**
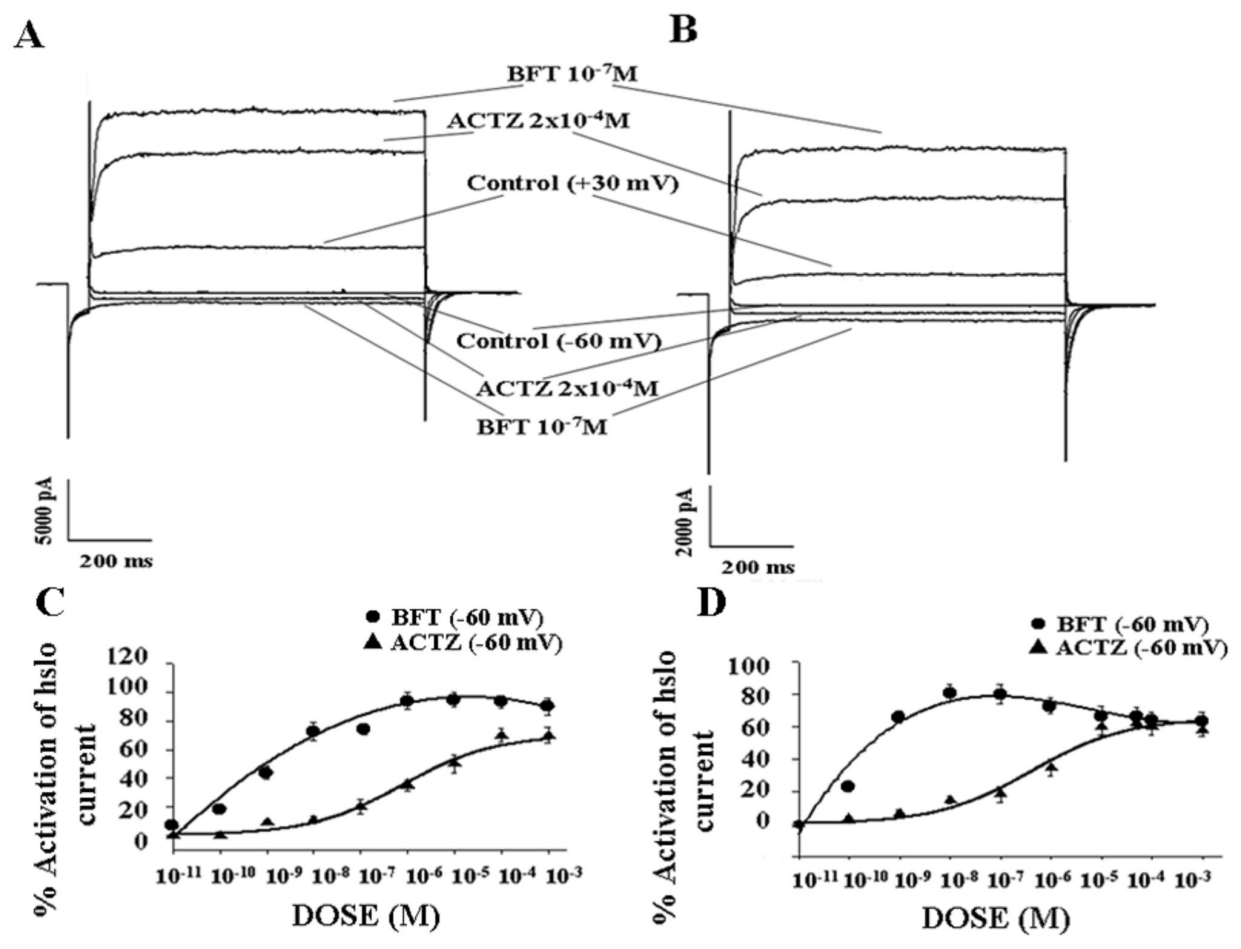
Effects of bendroflumethiazide (BFT) and acetazolamide (ACTZ) on hslo channel currents under hypokalemia and hyperkalemia conditions. Sample traces of macroscopic hslo channel currents recorded in HEK293 cells in the absence (control) or in the presence of BFT (10^-7^M) and ACTZ (2x10^-4^M), in the presence of 0.55 x10^-3^ M (A) or 15 x10^-3^ M (B) K^+^ ions in the bath and 150 x10^-3^ M K^+^ ions in the cell. The hslo channel currents were recorded in whole configuration in the presence of 10^-6^ M concentration of free Ca^2+^ ions in the cell using a voltage protocol: holding potential 0 mV; prepulse, -150 mV; test pulse, from -150 mV to + 150 mV in 10 mV steps; tail, -60 mV. The concentration–response relationships of BFT and ACTZ were constructed, at -60 mV (Vm), in the presence of 0.55 x10^-3^M (C) and 15 x10^-3^M (D) K^+^ ions in the bath and 150 x10^-3^ M K^+^ ions in the cell. Each experimental point represents the mean ± S.E. of the percentage activation of the hslo channel currents versus the compound concentrations of a minimum of three and a maximum of six patches. BFT was more potent than ACTZ in activating the hslo channel currents under hypokalemia and hyperkalemia conditions.

BFT and ACTZ enhanced hslo current in the presence of 0.55 x10^-3^ M and 15 x10^-3^ M concentrations of K^+^ ions in the bath solution with a potency and efficacy comparable with that observed in the presence of 5 x10^-3^ M K^+^ ions in the bath. The EC_50_ and Amax of ACTZ were 1.4+0.3 x10^-7^M and 72+3% (*n* slope=0.8), and 1.6+0.6 x10^-7^M and 69+7% (*n* slope=0.9) in the presence of 0.55 x10^-3^ M and 15 x10^-3^ M concentrations of K^+^ ions, respectively. The EC_50_ and Amax of BFT were 1.7+0.8 x10^-9^ M and 85+8% (*n* slope=0.8) and 1.9+0.8 x10^-9^ M and 84+9% (*n* slope=0.75) in the presence of 0.55 x10^-3^ M and 15 x10^-3^ M concentrations of K^+^ ions, respectively (Figure 5A, B).

**Figure 6:**
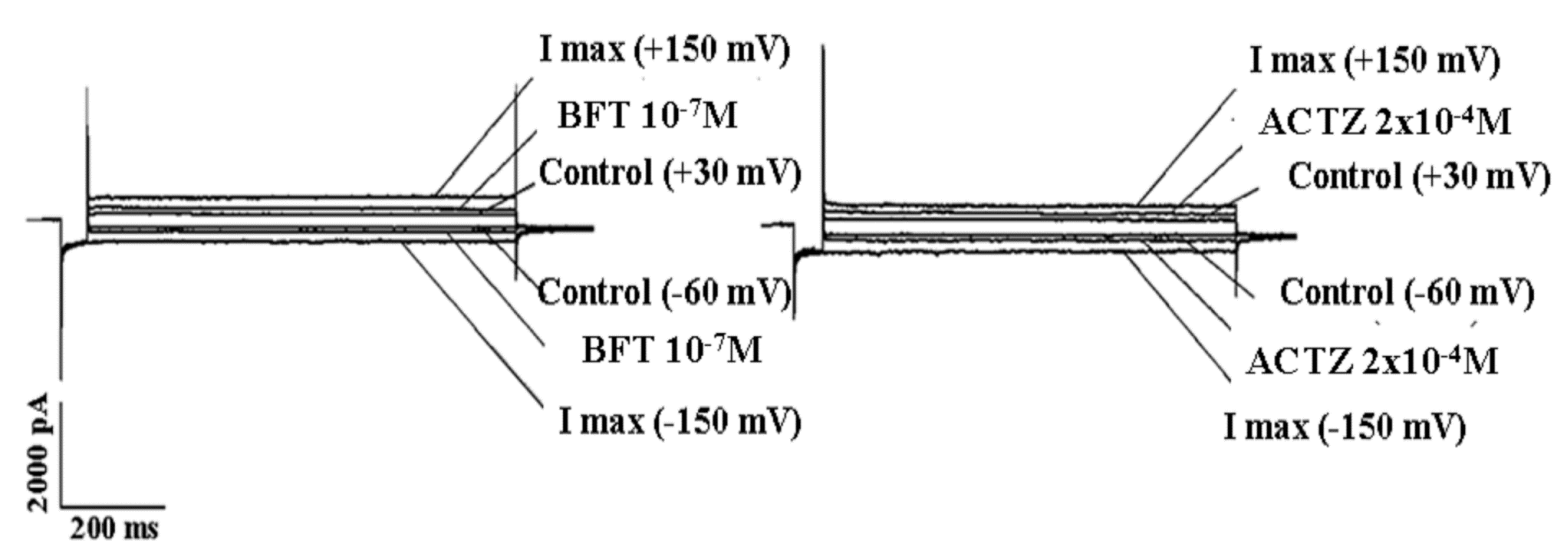
Effects of bendroflumethiazide (BFT) and acetazolamide (ACTZ) on currents recorded in not transfected HEK293 cells. The currents were recorded in the absence (Control) or presence of drugs, in the presence of 150 x10^-3^M and 5 x10^-3^M K^+^ ions in the cell and bath solutions, respectively, in the presence of 10^-6^ M concentration of free Ca^2+^ ions in the cell, using a voltage protocol: holding potential 0 mV; prepulse, -150 mV; test pulse, from -150 mV to + 150 mV in 10 mV steps; tail, -60 mV. BFT (10^-7^ M) and ACTZ (2x10^-4^M) in the same experimental conditions did not significantly affect the endogenous currents in not transfected HEK293 cells.

The exposure of the not transfected HEK293 cells to increasing K^+^ ion concentrations in the bath solution did not affect significantly the whole cell current (Number of cells=38). The effects of the BK openers and blockers on endogenous currents were also evaluated of not transfected HEK293 cells in the presence of 5 x10^-3^ M K^+^ ions in the bath solution. The exposure of the cells to BFT (10^-7^M) and ACTZ (2x10^-4^M), respectively, caused a 16.3±5% (Number of cells=6) and 9.3±3% % (Number of cells=9) enhancement of the current amplitude in respect to the controls that were not considered statistically significant (Figure 6). The other BK channel openers (2x10^-4^M) did not affect significantly the current amplitudes recorded in this cell line (Number of cells=23). The currents recorded in the HEK293 cells were not sensitive to ChTX (2x10^-7^M) or IbTX (4x10^-7^M) (Number of cells=13); TEA (5x10^-3^M) caused a -17+9% (Number of cells=10) reduction of the ion currents at +30 mV (Vm) which was not statistically significant.

### Changes of the Cell Viability Induced by K^+^ Ions Challenge in HEK293 Cell Lines and the Effects of the BK Channel Modulators

The effects of the hyperkalemia (K^+^ext. = 15 x 10^-3^M) and hypokalemia (K^+^ext. = 0.55 x 10^-3^M) on cell viability were investigated in HEK293 cells that were expressing the hslo subunit (hslo-HEK293) and in not transfected HEK293 cells. The effects of drugs and toxins on this parameter were tested at concentrations that were capable to fully block or activate the hslo channel in patch-clamp experiments.

**Figure 7:**
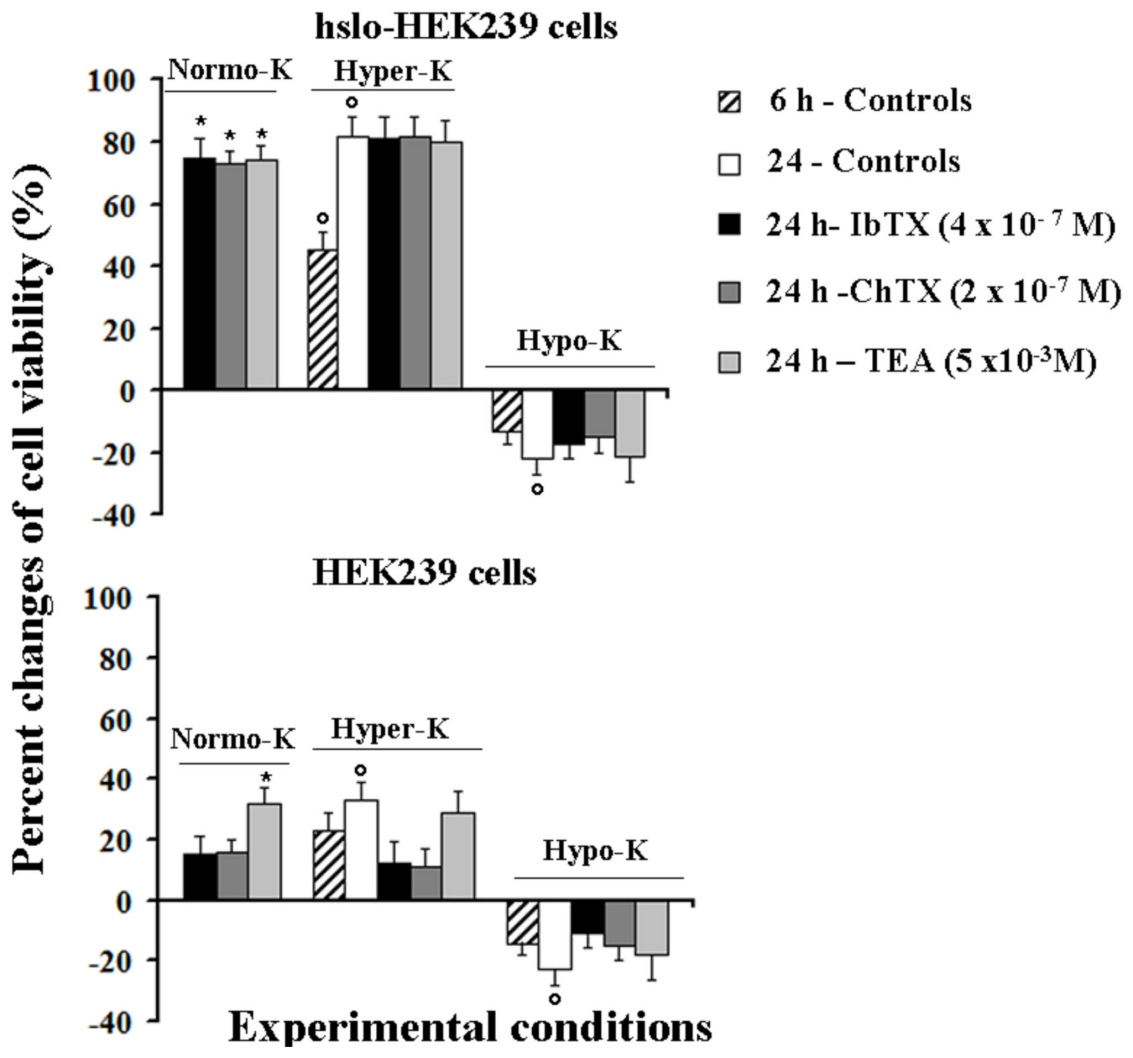
Effects of hyperkalemia and hypokalemia conditions on cell viability of HEK293 cells that were expressing the hslo subunit (hslo-HEK293) and pharmacological modulation by BK blockers. The cell viability was evaluated using the CCK-8 test in hslo-HEK293 and HEK293 cells after incubation for 6 h and 24 h, in the presence of 5 x 10^-3^M (Normo-K), 15 x 10^-3^M (Hyper-K) or 0.55 x 10^-3^M (Hypo-K) K^+^ ions in the external medium, in the absence of drugs (controls) or in the presence of iberiotoxin (IbTX), charybdotoxin (ChTX) and tetraethylammonium (TEA). The toxins and TEA enhanced the cell viability as well as the hyper-K conditions, while the hypo-K conditions reduced the cell viability. Data were significantly different between drug treatments and controls under hyperkalemia, hypokalemia or normokalemia conditions after 24 h of incubation time (*) as determined by using the two way analysis of variance (p<00.5). Data were significantly different in respect to the controls under normokalemia conditions (°) as determined by student t-test (p < 00.5).

The incubation of the hslo-HEK293 for 24 h either with TEA (5x10^-3^M), IbTX (4x10^-7^M) or ChTX (2x10^-7^M) in normokalemia (ext. K^+^ ions = 5 x 10^-3^M) conditions significantly increased the cell viability with respect to the controls (Figure 7). TEA was also effective in inducing proliferation of the not transfected HEK293 cells in the absence of hslo subunit, but IbTX and ChTX were not effective in this cell line. A significant enhancement of the cell viability was observed after 6 h and 24 h of incubation time under hyperkalemia in respect to the normokalemia conditions in the hslo-HEK293 cells. No further enhancement of this parameter was observed in the presence of the channel BK blockers in this cell line. These effects were more pronounced in the hslo-HEK293 cells in respect to the HEK293 cells. In contrast, the incubation of the cells for 6 h and 24 h in the presence of 0.55 x 10^-3^M concentrations of K^+^ ions caused a significant and comparable reduction of the cell viability in either hslo-HEK293 and HEK293 cells (Figure 7).

**Figure 8:**
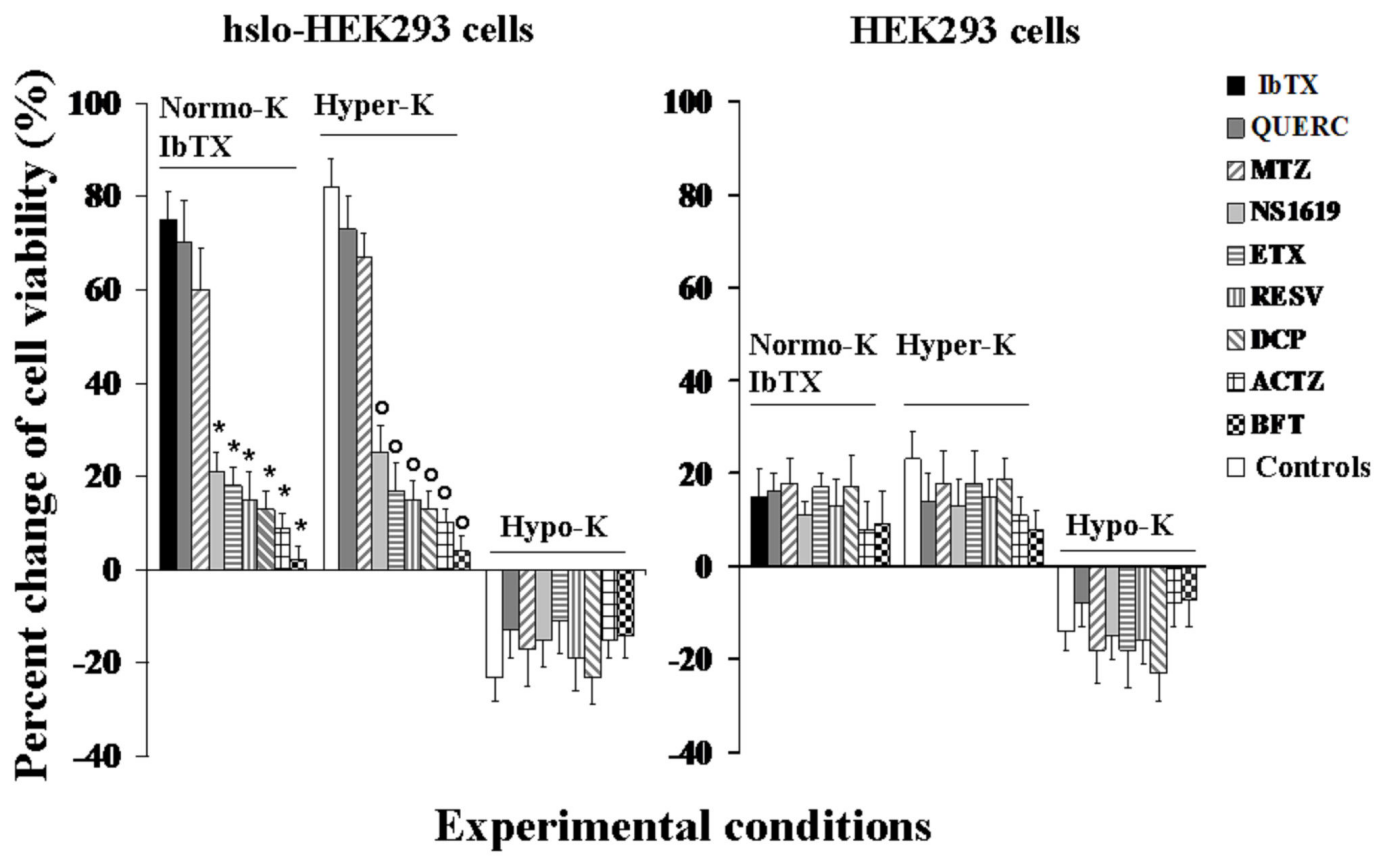
Effects of hyperkalemia and hypokalemia conditions on cell viability of HEK293 cells that were expressing the hslo subunit (hslo-HEK293) and pharmacological modulation by BK openers. The cell viability was evaluated using the CCK-8 test in hslo-HEK293 and HEK293 cells after incubation for 24 h, in the presence of 5 x 10^-3^M (Normo-K) +IbTX (4x10^-7^M), 15 x 10^-3^M (Hyper-K) or 0.55 x 10^-3^M (Hypo-K) K^+^ ions in the external medium, in the absence of drugs (controls) or in the presence of NS1619 (2x10^-4^ M), bendroflumethiazide (BFT) (10^-7^ M), acetazolamide (ACTZ) (2x10^-4^ M), dichlorphenamide (DCP) (2x10^-4^ M), methazolamide (MTZ) (2x10^-4^ M), ethoxzolamide (ETX) (2x10^-4^ M), quercetin (QUERC) (2x10^-4^ M) and resveratrol (RESV) (2x10^-4^ M). Data were significantly different between drug treatments and normokalemia+IbTX conditions (*) or hyperkalemia conditions (**_°_**) after 24 h of incubation time as determined by using the two way analysis of variance (p < 00.5).

BFT(1x10^-7^M), ACTZ(2x10^-4^M), DCP(2x10^-4^M), NS1619(2x10^-4^M), ETX(2x10^-4^M) and RESV (2x10^-4^M) were capable to restore to control values the observed increase of hslo-HEK293 cell viability induced by IbTX under normokalemia conditions or by hyperkalemia, while MTZ (2x10^-4^M) and QUERC(2x10^-4^M) were less effective (Figure 8). In the absence of the hslo subunit the BK openers did not significantly affect the cell viability in the presence of IbTX or hyperkalemia (Figure 8).

The efficacy ranking of the drugs in preventing the hyperkalemia-dependent increase of the cell viability based on the two way analysis of variance was BFT> ACTZ ≥ DCP≥ RESV ≥ ETX > NS1619 > MTZ≥ QUERC. These drugs showed an efficacy comparable to that observed under hyperkalemia conditions in preventing the IbTX-induced increase of the cell viability (Figure 8).

**Figure 9:**
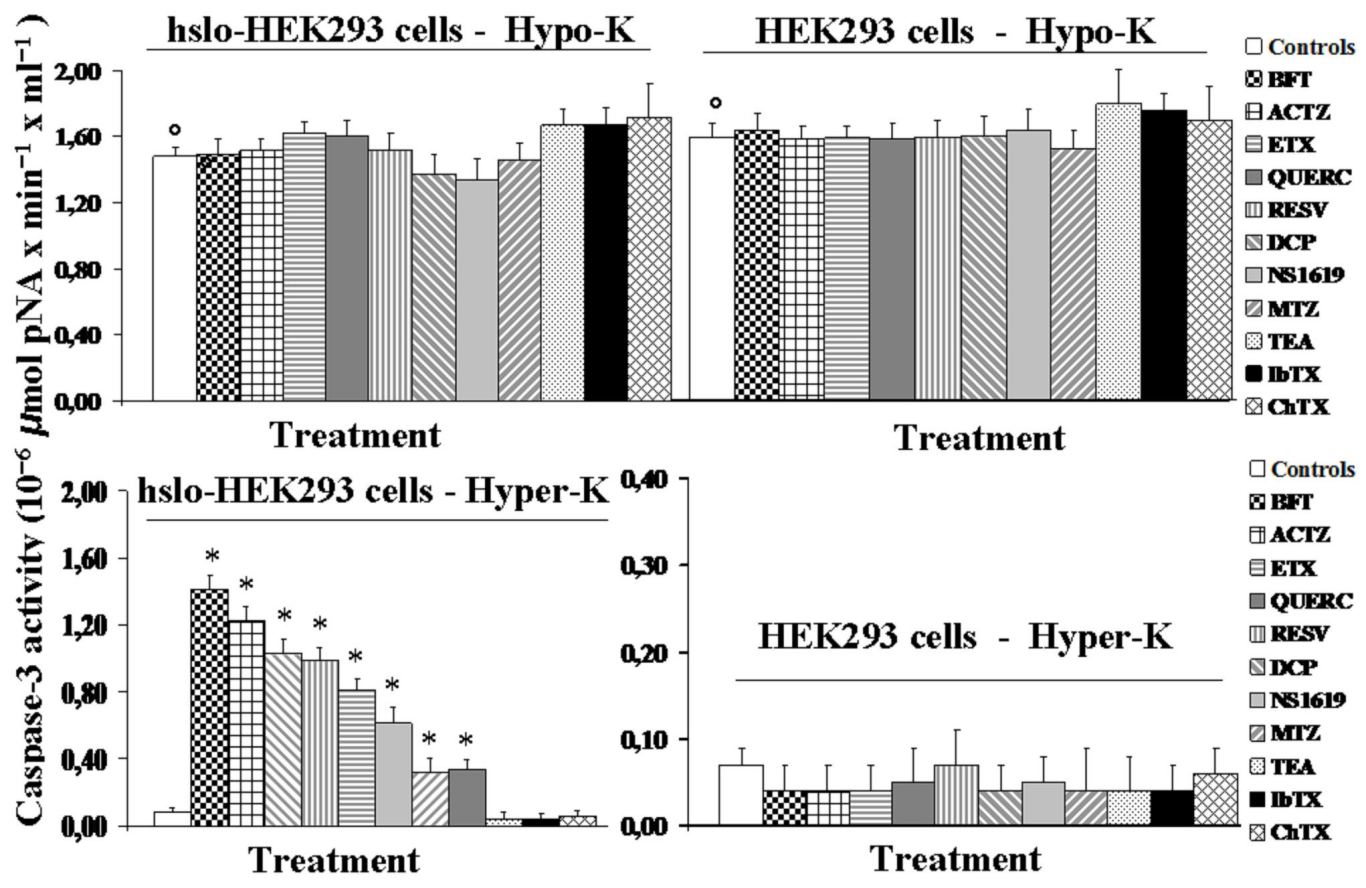
Effects of hyperkalemia and hypokalemia conditions on caspase-3 activity of HEK293 cells that were expressing the hslo subunit (hslo-HEK293) and pharmacological modulation by BK openers. The caspase-3 activity was evaluated in hslo-HEK293 and HEK293 cells after incubation for 24 h, in the presence of 15 x 10^-3^M (Hyper-K) or 0.55 x 10^-3^M (Hypo-K) K^+^ ions in the external medium, in the absence of drugs (controls) or in the presence of NS1619 (2x10^-4^ M), bendroflumethiazide (BFT) (10^-7^ M), acetazolamide (ACTZ) (2x10^-4^ M), dichlorphenamide (DCP) (2x10^-4^ M), methazolamide (MTZ) (2x10^-4^ M), ethoxzolamide (ETX) (2x10^-4^ M), quercetin (QUERC) (2x10^-4^ M) and resveratrol (RESV) (2x10^-4^ M). The drug treatments increased the caspase-3 activity under hyperkalemia conditions in hslo-HEK293 cells but not in HEK293 cells. Data were significantly different between drug treatments and hyperkalemia conditions (*) as determined by using the two way analysis of variance (p<00.5). The drug treatments failed to affect the caspase-3 activity under hypokalemia conditions in either hslo-HEK293 and HEK293 cells as determined by the two way analysis of variance (p<00.5). Data were significantly different in respect to the controls under normokalemia conditions (**_°_**) as determined by using the student-t test (p < 00.5).

A significant caspase-3 activity was measured after 24 h of incubation time under hypokalemia conditions in the cell lines under investigation. The caspase-3 activity was not significantly different between groups in hslo-HEK293 and in HEK293 cells. BK openers and blockers failed to prevent the hypokalemia-induced caspase-3 activation in the hslo-HEK293 and HEK293 cells (Figure 9).

However, an enhancement of the caspase-3 activity was measured after 24 h of incubation in the presence of BK channel openers under hyperkalemia conditions in hslo-HEK293cells (Figure 9). The absolute efficacy ranking of the drugs in increasing the caspase-3 activity under hyperkalemia conditions based on the two way analysis of variance was BFT> ACTZ > DCP≥ RESV ≥ ETX >NS1619 > QUERC ≥ MTZ.

No measurable caspase-3 activity was measured after 24 h incubation time in the hslo-HEK293 and HEK293 cells under normokalemia conditions. BK openers and blockers did not affect caspase-3 activity under normokalemia conditions in these cell lines.

## Discussion

Here we showed that the hslo subunit is responsible for the hyperkalemia-induced enhancement of the HEK293 cell viability thereby contributing to the 49% change of this parameter observed in this experimental condition. This is observed in the absence of beta subunits indicating that the pore forming subunit is the main molecular structure required for this effect. A channel blocking mechanism is responsible for the observed hyperkalemia-dependent increase of the cell viability as demonstrated by the finding that in our experiments the hyperkalemia conditions reduce the outward efflux of K^+^ ions flowing through the BK channel thereby shifting the reversal potential of the cells toward positive membrane potentials. In addition, in our experiments BK channel blockers targeting the hslo subunit, tested at concentrations that were capable to fully block the channel in patch-clamp experiments, also enhanced cell viability in HEK293 cells that were expressing the hslo subunit but not in those cells not expressing this subunit.

The BK channel openers restored the cell viability to control values under normokalemia conditions in the presence of IbTX and under hyperkalemia conditions in hslo-HEK293 cells and their effects were correlated with their efficacy in activating the BK channel currents. This is demonstrated by the fact that the rank order of absolute efficacy of the openers (BFT> ACTZ≥DCP≥ RESV≥ ETX > NS1619> MTZ≥QUERC) was comparable with their rank order of efficacy in restoring the cell viability in hslo-HEK293 cells under normokalemia conditions, in the presence of IbTX, and under hyperkalemia conditions (BFT> ACTZ ≥ DCP≥ RESV ≥ ETX > NS1619 > MTZ≥ QUERC).

The actions of the BK openers under hyperkalemia conditions were mostly mediated by the activation of the caspase-3 activity in hslo-HEK293 cells as demonstrated by the fact that the rank order of efficacy of these drugs in inducing caspase-3 activation in hslo-HEK293 cells (BFT> ACTZ > DCP≥ RESV ≥ ETX >NS1619 > QUERC ≥ MTZ) was similar with that observed as BK openers in the same cells. These findings can be explained by the fact that a persistent activation of BK channel by drugs significantly decreased the concentration of K^+^ ions due to a sustained K^+^ efflux from the cells. Thus, the resultant K^+^ loss leads to cell shrinkage (apoptotic volume decrease) and relieves its tonic suppression on caspase-3 or nuclease activity [28,29]. Caspase-3 activation induced by NS1619 or tamoxifen has been reported under hyperglycemia conditions in HEK293 cells that were expressing the hslo+beta 1 subunits and were correlated with their actions as BK channel openers [22].

However, the hslo subunit does not seem to have a significant role in the reduction of the cell viability observed under hypokalemia in our experiments and the BK openers failed to induce cytoprotection against the low K^+^ -dependent reduction of cell viability and caspase-3 activation either in the hslo-HEK293 and HEK293 cells.

### Pathophysiological and Pharmacological Implications

The hslo-dependent increase of the cell viability may have a role in the hypertrophic phenotype observed in disease conditions associated with hyperkalemia or abnormal K^+^ ion homeostasis. For instance, myotonia is linked to the loss of function mutation of the sarcolemma chloride channel or gain of function of the Na^+^ channels which lead to fibers hyperexcitability, myotonic contraction, after-discharge, paralysis and hypertrophic phenotype [9,19,20]. These phenomena are associated with an excessive efflux of K^+^ ions through voltage-dependent K^+^ channels which accumulates into the T-tubules of the fibers during trains of action potentials leading to paralysis [19,20]. This is demonstrated by the fact that de-tubulation of the fibers induced by osmotic shock reduces the accumulation of K^+^ ions into the t-tubule preventing the characteristic after-discharge, the depolarization and paralysis [19]. It may be possible that the BK channel block induced by the K^+^ ion accumulation into the t-tubule increases the cell viability leading to a hypertrophic phenotype observed in animal and human myotonic patients [9,19,20].

In our experiments, the hypokalemia conditions induced a significant reduction of the cell viability associated with caspase-3 activation that however appears to be independent of hslo expression. This mechanism may help to explain the permanent muscular atrophy and weakness observed in patients affected by hypoPP type -1 [15].

Here we showed for the first time that the BFT and ACTZ molecules were the most powerful drugs within the investigated compounds capable to activate the hslo channel and restore the cell viability to controls under hyperkalemia conditions. BFT is also an inhibitor of the renal thiazide sensitive co-transporter which support the use of this drug in the hyperkalemias form of periodic paralysis or in treating myotonia phenotype. BFT is capable to activate skeletal muscle KATP channels at micromolar concentrations thereby guarantee an efficient restoration of the resting potentials of the fibers. In the K-depleted rat, not a genetic animal model of hypokalemic periodic paralysis, BFT is effective in preventing the paralysis and weakness induced by insulin [26,27]. However, BFT showed a reduced efficacy in the activating the hslo channel in the micromolar concentration range. This finding can be explained assuming the existence of two different sites with different affinities for BFT and different capability to activate the channel. One mechanism can be related to the property of this channel to undergo desensitization following exposure of the cell to increasing concentrations of a drug. For instance, BK channel desensitization has been described following exposure of hslo channel expressed in HEK293 cell to alcohol [30].

ACTZ and DCP are used to treat various forms of periodic paralysis. The observed action of ACTZ and DCP on hslo channel at the negative membrane potential in the sub-micromolar concentration range suggests that this mode of action can be a pre-requisite for BK openers effective in PP, and explain their preferential use in reducing the attack frequency of PP rather than during the paralytic attack. The action of these drugs at the negative membrane potential of BK channel would indeed favor the fiber repolarization shifting the control of the resting potential under a voltage and Ca^2+^ -dependent current component. Opening of the BK channels by drugs is indeed one of the mechanism resolving the fiber depolarization in PP. The beneficial action of ACTZ on the cell viability under hyperkalemia conditions may help to explain the therapeutic effect of this drug in the acetazolamide-responsive myotonia also known as potassium aggravated myotonia which is indeed precipitated by K^+^ ions ingestion [18].

In conclusion, we believe that that activation/inhibition of skeletal muscle BK channel might be a mechanism regulating fibers remodelling by balancing cell apoptosis and proliferation under hyperkalemia conditions.

## Materials and Methods

### Heterologous Expression of hslo Subunit in Human Embryonic Kidney 293 (HEK293) Cells

Transient expression of human alpha-BK channels in HEK293 cells were achieved by 10 ng of plasmid DNA encoding the alpha subunit of the human BK channel using the calcium phosphate co-precipitation method [31]. The human full-length cDNA of Slo1 alpha subunit (U11058, *KCNMA1*) was subcloned in pcDNA 3.1 vectors (Invitrogen) using standard recombinant DNA techniques. This channel was co-transfected with plasmid DNA encoding CD8 receptors in a 10:1 plasmid mass ratio. Successfully transfected cells (hslo-HEK293) were identified using Dynal microbeads coated with anti-CD8 antibody (Dynal A.S., Oslo, Norway) and were used for patch-clamp experiments or cell viability investigations 36–96 h after transfection.

### Recording Solutions and Drugs

In whole cell experiments, the pipette (intracellular) solutions contained (10^−3^M): 150 KCl, 5 ethylene glycol bis (β-aminoethyl ether)-N, N, N, N*-*tetraacetic acid (EGTA), 10 MOPS sodium salt, pH= 7.2 with MOPS acid. The bath solution contained (10^−3^M) : 0.55, 5, 15 or 150 KCl, 5 ethylene glycol bis (β-aminoethyl ether)-N, N, N, N*-*tetraacetic acid (EGTA), 10 MOPS sodium salt, pH= 7.2 with MOPS acid. Osmolarity was adjusted by adding sucrose to the bath solution and ranged from 280 to 306 mosmol/l. The addition of the N-methyl D-glucamine to the solutions was avoided due to the interference of this substance with BK channel currents [32]. CaCl_2_ was added to the pipette and bath solutions to give free Ca^2+^ ion concentration of 10^−6^ M in whole cell experiments. The calculation of the free Ca^2+^ ion concentration in the pipette was performed as described previously [25,26].

The BK openers under investigations were : NS1619, ACTZ, DCP, MTZ, ETX, HCT, BFT, QUERC, RESV (Figure 1A, B, C). The selective BK blockers investigated here were iberiotoxin (IbTX) and charybdotoxin (ChTX), the unselective blocker was tetraethylammonium (TEA). The drugs and toxins were purchased from Sigma (Sigma Chemical Co., Mi) Stock solutions of the drugs under investigation were prepared dissolving the drugs in dimethylsulphoxide (DMSO) at concentrations of 20 x 10^−3^ M. Microliter amounts of the stock solutions were then added to the bath solutions as needed. DMSO did not exceed 0.01% in the bath, at this concentration the solvent does not normally affects the hslo channel currents.

For cell viability experiments the composition of the bath solutions were the normal Ringer solution (normo-K) : 145×10^−3^ M NaCl, 5×10^−3^ M KCl, 1×10^−3^ M MgCl_2_, 0.5×10^−3^ M CaCl_2_, 5×10^−3^ M glucose and 10×10^−3^ M 3-(*N-*morpholino) propanesulfonate (Mops) sodium salt and was adjusted to pH 7.2 with Mops acid; the hyperkalemic solution (hyper-K) was a Ringer solution enriched with KCl: 15x10^-3^M KCl; the hypokalemic solution (hypo-K) was a Ringer solution depleted of KCl: 0.55x10^-3^M KCl. Osmolarity was adjusted by adding sucrose to the solutions and ranged from 280 to 306 mosmol/l.

### Patch-clamp experiments

The hslo currents and drug actions on the hslo channel currents expressed in the HEK293 cells (hslo-HEK293) were investigated during voltage steps using whole cell of patch-clamp technique in the presence of internal 10^-6^M of free Ca^2+^ ion concentrations. Complete concentration–response relationships analysis were performed at -60 mV and + 30 mV (Vm) for all drugs on the whole-cell channel currents in the presence of 5 x 10^-3^M KCl in the bath solution and 150 x10^-3^M KCl in the cell. BFT and ACTZ were also tested on hslo current in the presence of 0.55 x10^-3^M, 15 x10^-3^M and 150 x10^-3^M KCl in the bath solution; while the intracellular solution contained 150 x10^-3^M KCl.

The hslo currents were recorded at 20° C and sampled at 1 kHz (filter=2 kHz) using an Axopatch-1D amplifier equipped with a CV-4 headstage (Axon Instruments, Foster City, CA). The voltage protocol was: holding potential 0 mV, prepulse - 150 mV(duration=80 ms), test pulse from -150 mV to +150 mV in 10 mV steps(duration=600 msec), tail - 60 mV(duration=250 ms). The channel currents were identified on the basis of their voltage dependence, current amplitude, response to toxins and drugs (33–35). The mean hslo channel currents in the presence of 150 x10^-3^ M KCl in the bath and in the cell, leak subtracted, were -480+51 pA, -403+37 pA, 3201+ 97 pA and 7000+ 100 pA at -150 mV, -80 mV, +80 mV, and +150 mV (N of cells = 235) of membrane potentials, respectively. The application of 4x10^-7^ M and 2x10^-7^ M concentrations of IbTX or ChTX produced a significant reduction of the channel current at positive membrane potentials.

Current analysis was performed using pClamp 10 software package (Axon Instruments). The criteria for accepting the data entering were based on the stability of the seal evaluated by observing the noise levels not exceeding 0.6 pA at 2 kHz. No correction for liquid junction potentials was made, which were estimated to be <1.9 mV under our experimental conditions. Pipettes resistance was 2.3+0.2 MΩ (Number of pipettes=275).

The applied protocol for drug response in whole-cell patch experiments were as follows:

1 internal (pipette) KCl(150x10^-3^M) +Ca^2+^ ions (10^-6^ M) /

external (bath) KCl (0.55, 5, 15 or 150 x10^-3^M) +Ca^2+^ ions (10^-6^ M) control

2 internal KCl (150x10^-3^M) + Ca^2+^ions (10^-6^ M) /

external KCl (0.55, 5, 15 or 150 x10^-3^M) + Ca^2+^ions (10^-6^ M) drug

3 internal KCl (150x10^-3^M)+Ca^2+^ions (10^-6^ M) /

external KCl (0.55, 5, 15 or 150 x10^-3^M) +Ca^2+^ions (10^-6^ M) washout

4 internal KCl (150x10^-3^M)+Ca^2+^ions (10^-6^ M) /

external KCl (0.55, 5, 15 or 150 x10^-3^M) +Ca^2+^ions (10^-6^ M) drug + IbTX (4x10^-7^M) or ChTX (2x10^-7^M)

The cells were exposed to the drug solutions for 10 sec before recordings. Increasing concentrations of drug solutions were applied to the cells by the fast perfusion system (AutoMate, Sci. U.S.A.). Each application of drug solution was followed by a washout period of 6 s to allow recovering of channel currents to control values. No more than three different drug concentrations were applied to the same cell, with one compound per cell tested at a time. Due to the not reversibility of the QUERC action following washout during the time of observation, only one concentration per cell and plate was tested at a time for this drug. Seal resistance was continuously monitored during patch solutions exchange. At the end of the drug protocol test, the cells were exposed to a bath solution enriched with IbTX (4x10^-7^M) or ChTX (2x10^-7^M).

Drug experiments were also performed to evaluate possible activation or inhibition of endogenous channel currents by drugs. The drugs were tested against the currents of not transfected HEK293 cells using the same drug protocol test as above described.

### Cell viability assay and caspase 3 activity measurements

Cell viability was evaluated by measuring the succinic dehydrogenases activity in the cell suspension using the cell Counting Kit-8 (Enzo Life Sciences International, Inc, U.S.A.) which utilizes highly water-soluble tetrazolium salt. WST-8 2-(2-methoxy-4-nitrophenyl)-3-(4- nitrophenyl)-5-(2,4-disulfophenyl)-2H-tetrazolium, monosodium salt] produces a water-soluble formazan dye upon reduction in the presence of an electron carrier. It is reduced by dehydrogenases in cells to give a yellow colored product (formazan), which is soluble in the tissue culture medium. The detection sensitivity of CCK-8 is higher than other tetrazolium salts. The changes of the cell vitality were expressed as % changes of cell viability induced by hyper-K^+^ or hypo-K^+^ conditions with respect to the controls in the presence or absence of drugs.

Caspase-3 activity was measured in the cell suspension using a colorimetric assay based on the hydrolysis of the peptide substrate acetyl-Asp–Glu–Val–Asp-*p*-nitroaniline (Ac-DEVD-pNA) by caspase-3 resulting in the release of the pNA which has a high absorbance at 405 nm. The reagents and the CASP-3C kit used were supplied by Sigma (Milano).

Hslo-HEK293 cells and not transfected HEK293 cells were incubated with hyper-K, hypo-K or normo-K solutions, in the presence or absence of drugs for 6 h or 24 h under 5% CO_2_-95% O_2_ atmosphere for the maintenance of aerobic conditions, at 37^◦^C.

## Data analysis

The data are expressed as mean + S.E. unless otherwise specified.

The concentration–response relationships of the openers under investigation could be fitted with the following equations:

(*I* drug – *I* control/*I* control-*I* max) x 100 = *A* max_a_/(1+EC_50a_/Drug)^*na*^ (1)

(*I* drug – *I* control/*I* control-*I* max) x 100 = *A* max_b_/(1+ Drug/EC_50b_)^*nb*^ (2)

For all drugs under investigation the data were fitted by using equation 1. In the case of BFT molecule that enhanced the channel currents at low concentrations and showed a reduced capability to enhance the currents at higher concentrations the data were fitted using the sum of equation 1 and equation 2. *I* drug is the hslo- channel current measured in the presence of the molecules under study and normalized to the maximal currents recorded in the same patches; *A* max_a_ and *A*max_b_ are the percentage of maximal activation of the BK currents produced by the molecules under study; EC_50a_ and EC_50b_ are the concentrations of the drugs needed to enhance the current by 50%; Drug is the concentration of the drug tested; *I* max is the maximal current recorded in the patches at 150 mV or -150 mV (Vm); *I* control is the current recorded in the absence of drugs; and *n*_*a*_ *or n*_*b*_ are the slope factors of the curves. The algorithms of the fitting procedures used are based on a Marquardt least-squares fitting routine. Data analysis and plot were performed using SigmaPlot software (Systat Software, Inc., San Jose, CA). The potency ranking of the openers expressed as EC_50a_ was constructed comparing the EC_50a_ data of two drug treatments using the student t-test, differences between mean were evaluated at p<0.05 level of significance. The symbol > was assigned in case of a drug showing a major absolute EC_50a_ value but not statistically significant, while the symbol > was assigned when the calculated EC_50a_ values were statistically significant in respect to those calculated for other drugs.

The variance analysis was used for a multiple comparison of statistically significant differences between all drug treatments in the same range of concentrations, and the sample sizes (number of data points) used to calculate the degree of freedom were similar between groups in our experiments. The variance ratio is calculated as: mean square of the drug treatments / mean square of the residuals. This value is then compared with that tabulated at a certain degree of freedom at p<0.05 level of significance. A variance ratio larger than that tabulated indicates a rejection of the null hypothesis and a significant treatment effect. The absolute efficacy ranking of the drugs as BK openers was based on the one way analysis of variance that compare the EC_50_ values, A max and threshold concentrations of the drugs. The efficacy ranking of the drugs to prevent the hyperkalemia or hypokalemia –dependent changes of the cell viability and caspase 3-activity was based on the two way analysis of variance. The symbol > was assigned in case of a drug showing a major absolute value but not statistically significant, while the symbol > was assigned when the calculated values were statistically significant in respect to those calculated for other drugs.

The % change of the cell viability induced by the external K^+^ ions challenge (hyper-K or hypo-K) was calculated in respect to the normokalemia conditions; in the presence of BK channel blockers, it was calculated in respect to the control conditions (absence of blockers) using the following equations:

% change of the cell viability = (Hyper-K or Hypo-K / Normo-K) x 100;

% change of the cell viability = ((Normo-K+ Blockers) / Normo-K) x 100.

The effects of the BK channel openers on the cell viability were evaluated vs the changes of this parameter induced by IbTX under normokalemia conditions (Normo-K + IbTX), hyperkalemia (Hyper-K) or hypokalemia (Hypo-K) conditions using the following equations:

% change of the cell viability = ((Normo-K + IbTX) + Openers) / (Normo-K + IbTX) x 100;

% change of the cell viability = ((Hyper-K or Hypo-K) + Openers) / (Hyper-K or Hypo-K) x 100.
